# Nutritional risk assessment and nutritional support in children with congenital diabetes during surgery

**DOI:** 10.1515/med-2025-1204

**Published:** 2025-06-26

**Authors:** Jiaoyan Lian, Fang Zhang, Haoyu Chen

**Affiliations:** Department 1 of Pediatric Digestive, Gansu Provincial Maternity and Child-care Hospital, Lanzhou 730050, Gansu Province, China; Department 2 of Pediatric Digestive, Gansu Provincial Central Hospital, Lanzhou 730070, Gansu Province, China

**Keywords:** children with congenital diabetes, nutritional risk, nutritional support

## Abstract

**Objective:**

This work investigated intraoperative nutritional risk and nutritional support for children with congenital diabetes.

**Materials and methods:**

A total of 60 cases of children with congenital diabetes were assigned into two groups: the intravenous group (*n* = 30) and the oral group (*n* = 30). Medications were administered based on the actual conditions of patients. After surgery, the relationship between intraoperative nutritional risk score and glucose changes was studied.

**Results:**

All patients had normal liver and kidney function. In the intravenous group, 26 cases had sufficient calorie intake. The numbers of cases with normal nutrition, malnutrition, and severe malnutrition were 7, 12, and 11, respectively. The incidences of infection, delayed wound healing (DWH), difficult glucose control (DGC), and respiratory system complications were 3, 6, 12, and 1, respectively. In the oral group, 21 cases had sufficient calorie intake. Numbers of cases with normal nutrition, malnutrition, and severe malnutrition were 8, 9, and 13, respectively. The incidences of infection, DWH, and DGC were 2, 6, and 9, respectively. Fasting blood glucose <6.5 mmol/L and body mass index <17.3 were independent risk factors affecting nutritional support.

**Conclusion:**

Nutritional risk assessment and support during surgery for children with congenital diabetes were crucial for appropriate support.

## 1 Introduction

Children with congenital diabetes are a rare genetic condition characterized by symptoms of high blood sugar present at birth [1,2]. This condition not only impacts the growth and development of affected children but also has a significant influence on their nutritional intake [3,4]. Due to the inability of their bodies to properly utilize glucose, these children experience a restricted energy supply, resulting in slow weight gain and even malnutrition [5]. During surgery, special attention needs to be given to the nutritional status of these children. The surgery itself places a significant demand on the body, and children with congenital diabetes are more prone to energy deficiencies due to their metabolic abnormalities [6]. Therefore, assessing the nutritional risk of these children and providing appropriate nutritional support becomes an essential preoperative task.

Assessing the nutritional risk in children with diabetes requires considering multiple factors, including the child’s age, physical condition, and the type of surgery [7]. Age is an important factor because children in different age groups have varying nutritional needs. The physical condition of the child also needs to be taken into account, such as whether they have other complications or malnutrition. The type of surgery can also affect the nutritional requirements of a child, as some surgeries may impair digestive function, impacting the child’s ability to absorb and utilize nutrients [8]. Based on the assessment results, doctors can develop personalized nutritional support plans to ensure that the child receives adequate nutrition during surgery. This may involve increasing energy and protein intake to meet the child’s growth and repair needs [9,10]. Additionally, supplementation with vitamins and minerals may be considered to enhance the child’s immune function and recovery capacity. Before surgery, a comprehensive assessment of the nutritional status of the child is necessary, including measurements of height, weight, body fat content, and other indicators to understand his or her overall nutritional status. Dietary habits and intake should also be assessed to determine the risk of malnutrition [11]. There are various methods of nutritional support, which can be provided through oral or intravenous administration. For children who can eat, nutritional intake can be improved by adjusting the diet structure and increasing energy density. For children who cannot eat, special formula nutritional solutions can be administered intravenously to meet their nutritional needs [12]. Additionally, attention should be paid to the child’s intake of trace elements and vitamins. Due to the metabolic abnormalities in children with congenital diabetes, their requirements for certain trace elements and vitamins may be increased. Therefore, while providing nutritional support, it is important to consider supplementing these nutrients to maintain the child’s health.

Although perioperative nutritional management strategies are well-established for the general pediatric population, the unique characteristics of children with congenital diabetes (such as insulin secretion deficiencies and metabolic instability) result in significantly different nutritional requirements. Currently, research in this population has primarily focused on glucose control, while systematic assessments of perioperative nutritional risks and support strategies are extremely limited [13]. In particular, there is a lack of evidence-based nutritional intervention protocols, which leads to reliance on empirical decision-making in clinical practice, potentially increasing the risk of postoperative complications. Therefore, this study aimed to identify the perioperative nutritional risk characteristics in children with congenital diabetes through a retrospective analysis and explore clinical pathways for optimizing nutritional support, thereby addressing the knowledge gap in this field.

## 2 Materials and methods

### 2.1 Experimental design

A retrospective analysis was conducted on 60 cases of children with congenital diabetes who were treated at Gansu Maternal and Child Health Hospital between January 2019 and December 2021. Initially, a prospective study design was considered to minimize recall and selection biases. However, due to the rarity of congenital diabetes and the ethical limitations associated with obtaining timely prospective informed consent during emergency surgical interventions, a retrospective approach was adopted. While this method enables effective data collection, it acknowledges the limitations, such as missing records, incomplete dietary logs, and potential inaccuracies in retrospective blood glucose measurements. To mitigate these biases, strict inclusion/exclusion criteria were applied, and only cases with complete medical records were included.

Gastrointestinal surgeries (such as enterostomy and appendectomy, *n* = 22); endocrine-related surgeries (such as partial pancreatectomy, *n* = 18); and other non-gastrointestinal surgeries (such as hernia repair and orthopedic surgeries, *n* = 20). The complexity of the surgeries was classified according to the *Pediatric Surgical Risk Assessment Guidelines* (Clavien–Dindo classification): Grade I (minor injury, *n* = 35), Grade II (moderate injury, *n* = 25). Before the surgery commenced, fasting venous blood (FVB) samples were taken from the children for routine blood tests, liver function tests, and kidney function tests. Based on the method of nutritional support, the children were assigned to an intravenous group (*n* = 30) and an oral group (*n* = 30).

Throughout the study, all children underwent comprehensive examinations and nutritional risk assessments. Their nutritional risk assessment scores and glucose changes during the surgery were recorded based on dietary brief analysis, and medications were administered according to each child’s individual condition. After the surgery was completed, the glucose changes in the children were documented and organized, and the relationship between these glucose changes and the nutritional risk assessment during the surgical period was investigated.

### 2.2 Criteria for enrolling and excluding the study subjects

Criteria for enrolling and excluding the study subjects are outlined in Tables 1 and 2, respectively.

**Table 1:** Criteria for participant inclusion

No.	Descriptions
1	Aged 0–12 years old
2	Diagnosed with congenital diabetes
3	In need of surgical treatment
4	Provided informed consent from children and their legal guardians

**Table 2:** Criteria for participant exclusion

No.	Descriptions
1	Severe complications requiring emergency intervention, including: heart failure (NYHA classification III–IV), renal insufficiency (eGFR < 30 mL/min/1.73 m^2^), and active infections (CRP > 50 mg/L requiring intravenous antibiotic therapy)
2	Comorbid chronic conditions include: malignant tumors (such as leukemia, lymphoma, diagnosed according to WHO ICD-10 criteria), chronic liver disease (Child-Pugh classification B or C), and autoimmune diseases (such as systemic lupus erythematosus, meeting ACR diagnostic criteria)
3	Inability to receive nutritional support (e.g., intestinal obstruction, severe swallowing dysfunction)
4	Incomplete medical records (e.g., missing preoperative blood glucose data or nutritional assessment information)

### 2.3 Scales for assessing intraoperative nutritional risk

This study utilized the pediatric perioperative nutritional risk assessment (PPNRA) scale to evaluate the nutritional risk in children. Although the PPNRA scale has been validated for its reliability and validity in general pediatric surgical patients [14,15], specific validation studies for children with congenital diabetes have not been reported. Therefore, the application of the PPNRA scale in this study may have limitations, such as not fully covering diabetes-related metabolic risk factors (e.g., insulin resistance, blood glucose variability). To minimize bias, additional blood glucose control indicators (such as HbA1c) were recorded during the assessment to assist in the evaluation. The steps involved in using the PPNRA scale were as follows:

(1) Collecting basic information: basic information about the children was gathered, including age, gender, height, weight, and other relevant details.
(2) Assessing nutritional status: the standard nutritional assessment methods were employed, such as body mass index (BMI), height-weight percentiles, and other relevant metrics, to evaluate the nutritional status of the children.
(3) Evaluating surgical risk: the surgical risk level for each child based on the type of surgery and the level of surgical risk.
(4) Using the PPNRA scale: the nutritional risk assessment was performed using the PPNRA scale based on the basic information, nutritional status, and surgical risk level of child.
(5) Interpreting assessment results: the assessment results were interpreted to determine whether the child was at risk of malnutrition. Typically, assessment results were categorized into three levels: low, moderate, and high.
(6) Developing nutritional intervention plans: based on the assessment results, appropriate nutritional intervention plans were formulated. High-risk children may require nutritional support or supplementation.
(7) Monitoring and adjustment: during the surgical period, it should regularly monitor the nutritional status of children and make adjustments as necessary to ensure they receive the appropriate nutritional support.

By following these steps and using the PPNRA scale, healthcare professionals can effectively assess the nutritional risk of children with congenital diabetes during the perioperative period and implement tailored nutritional interventions to optimize their health outcomes.

### 2.4 Dietary survey of children

A 24-h dietary recall method was used to survey the children’s dietary intake. Trained nutritionists conducted face-to-face interviews with the caregivers to record a detailed description of all foods consumed in the past 24 h (including food types, weights, and cooking methods). To reduce recall bias, a 3-day food frequency questionnaire (FFQ) was also employed, focusing on the frequency of intake of high-sugar and high-protein foods during the week prior to surgery. Additionally, the accuracy of energy intake was validated by comparing the dietary records with biochemical indicators (such as serum prealbumin and retinol-binding protein). This involved detailed recording of the dietary intake of children over a 24-h period, including the types of foods, their weights, and quantities consumed during each meal. Additionally, meal times and frequencies were noted. Subsequently, the nutritional elements in the children’s diet were analyzed. This analysis encompassed energy (calories), protein, fat, carbohydrates, vitamins, minerals, and other nutrients. To assess whether the nutritional intake of the children met their requirements, the intake of each nutrient was calculated and compared to their recommended daily intake. For determining calorie intake adequacy, if the intake was less than 20% of the standard requirement, it was considered insufficient. The standard calorie intake was calculated by multiplying the calorie standard supply per kilogram of body weight by the ideal weight of the children.

### 2.5 Observation parameters

#### 2.5.1 BMI

BMI is an indicator used to assess whether a person’s weight is healthy. The height and weight of children were collected, and their BMIs were calculated. The BMI was calculated with weight (in kilograms) divided by the square of height (in meters). According to the classification standards of the World Health Organization (WHO), the range of BMI can be categorized into: BMI < 18.5: underweight, BMI = 18.5–24.9: normal weight, BMI = 25–29.9: overweight, and BMI ≥ 30: obesity.

#### 2.5.2 Nutritional status

Nutritional status assessment is an important medical process used to evaluate a child’s overall health status, especially factors related to nutrition [16]. By analyzing the age, gender, height, weight, and other factors, the basal metabolic rate and activity level of children were calculated to determine their daily energy requirements. BMI scores were then employed to assess the physical condition of the child. Furthermore, dietary habits of the children were analyzed to evaluate whether their intake of various nutrients met the recommended levels, including protein, carbohydrates, fats, vitamins, minerals, and more. Based on the assessment information, an overall description of the nutritional status of the children was provided, and the appropriate nutritional support method during surgery was determined.

#### 2.5.3 Liver function

Approximately 5 mL of FVB was collected, with 2 mL used for complete blood count testing and 3 mL for serum separation. The serum separation process involved allowing the blood sample to stand at room temperature for 30 min, followed by centrifugation at 3,000 rpm for 10 min to collect the upper serum layer. Serum samples were immediately frozen and stored at −80°C for subsequent testing. First, the liver function tests included the examination of markers such as alanine aminotransferase (ALT), aspartate aminotransferase (AST), total bilirubin, direct bilirubin, alkaline phosphatase (ALP), and other indicators. Second, the liver function enzyme profile comprised tests for markers such as ALT, AST, ALP, and gamma-glutamyl transferase. Prior to surgery, an ultrasound examination was conducted to assess the child’s liver. Using an ultrasound probe, the child’s abdominal area was examined, and ultrasound images were used to observe the morphology, size, structure, and blood flow of the liver. This examination provided initial insights into the functional status of the liver and the presence of any abnormalities such as tumors or stones. Postoperative blood glucose monitoring time points were based on the *Pediatric Perioperative Glycemic Management Guidelines* [17] and clinical practice standards: venous blood samples were collected to measure fasting blood glucose (FBG) and postprandial blood glucose (PBG) at 0 h (post-anesthesia recovery), 6, 12, 24, and 48 h after surgery. These time points were selected to encompass the metabolic changes during the acute stress phase (0–24 h) and recovery phase (24–48 h) post-surgery while minimizing additional trauma to the children from frequent blood sampling.

#### 2.5.4 Kidney function

Midstream urine samples were collected from children, and urine creatinine (Ucr), blood urea nitrogen (BUN), and urine albumin-to-creatinine ratio (UACR) were measured using an automated biochemical analyzer (BS-240E, Shenzhen Mindray Bio-Medical Electronics Co., Ltd., China). FVB was also collected to measure serum creatinine (Scr) and cystatin C (Cys-C). Kidney function was assessed according to the *Pediatric Nephrology Diagnosis and Treatment Guidelines* (2020 edition):

Normal ranges: Scr (<1 year: 10–30 μmol/L; 1–12 years: 20–50 μmol/L); BUN (1.8–6.4 mmol/L); and UACR (<30 mg/g).

Abnormal values were defined as: Scr or BUN exceeding the age-specific upper limit, or UACR ≥ 30 mg/g.

#### 2.5.5 Biochemical testing

Biochemical testing is a common medical diagnostic method used primarily to assess the state of human health and diagnose diseases (19). The operational steps were as follows:

1. Preprocessing of samples: Depending on the testing requirements, serum and urine samples from the child were subjected to preprocessing, which involved precipitating and washing the substances to be tested.
2. Reagent preparation: Based on the testing method and sample characteristics, appropriate reagents were prepared. The samples were mixed with the reagents in the specified proportions to induce specific biochemical reactions.
3. Reaction termination and analysis: A termination solution was added to stop the reaction, and the reaction products were analyzed using a spectrophotometer. The testing results were then derived based on the data obtained from the analysis.

### 2.6 Methods for statistical analysis

Data were statistically analyzed using SPSS 26.0. Continuous data were expressed as (*x* ± *s*), and intergroup comparisons were conducted using independent sample *t*-tests. Categorical data were analyzed using the chi-square test (*χ* ^2^) or Fisher’s exact test (when expected frequencies were <5). A multifactorial analysis of variance was conducted for all postoperative complications (infection, delayed wound healing [DWH], difficult glucose control [DGC], respiratory system complications [RSC]), with group (intravenous/oral) and surgery type as fixed factors, while controlling for covariates such as age and BMI. When multiple factors were involved, multivariable logistic regression analysis was performed. The initial variables included FBG, BMI, surgery duration (minutes), intraoperative blood loss (mL), and postoperative complications (infection/delayed healing). Independent influencing factors were selected using stepwise regression (forward method, *α* = 0.1). *P* < 0.05 indicated a statistically great difference.


**Informed consent:** Informed consent was obtained from all individual participants included in the study.
**Ethical approval:** This study was performed in line with the principles of the Declaration of Helsinki.

## 3 Results

### 3.1 Basic characteristics of children

The basic characteristics of enrolled children are outlined in Table 3. It suggested that the age, weight, and gender of children were not greatly different (*P* > 0.05).

**Table 3:** Basic characteristics of children

Group	Age	Gender		Weight
		Male	Female	
Intravenous group	9 ± 3.63	17	13	29.81 ± 8.34
Oral group	9 ± 4.01	16	14	31.42 ± 9.13

### 3.2 Dietary survey results of children in different groups

The 24-h calorie intake between children in different groups was compared in Table 4. In the intravenous group, 26 cases (86.67%) had sufficient calorie intake, while 4 cases (13.33%) had insufficient intake. In the oral group, 21 cases (70%) had sufficient calorie intake, while 9 cases (30%) had insufficient intake.

**Table 4:** Comparison on the 24-h calorie intake of children

Group	Sufficient intake	Insufficient intake
Intravenous group	26 (86.67%)	4 (13.33%)
Oral group	21 (70%)	9 (30%)*

^*^Suggested a statistically remarkable difference with *P* < 0.05.

### 3.3 The nutritional status of diabetic children

The nutritional status of diabetic children in distinct group was presented in Table 5. In the intravenous group, there were 7 cases (23.33%) with normal nutrition, 12 cases (40%) with malnutrition, and 11 cases (36.67%) with severe malnutrition. In contrast, there were 8 cases (26.67%) with normal nutrition, 9 cases (30%) with malnutrition, and 13 cases (43.33%) with severe malnutrition in the oral group. These data exhibited no great difference (*P* > 0.05).

**Table 5:** The nutritional status of diabetic children in distinct groups

Group	Normal nutrition	Malnutrition	Severe malnutrition
Intravenous group	7 (23.33%)	12 (40%)	11 (36.67%)
Oral group	8 (26.67%)	9 (30%)	13 (43.33%)

### 3.4 Results of biochemical testing

**Figure 1:**
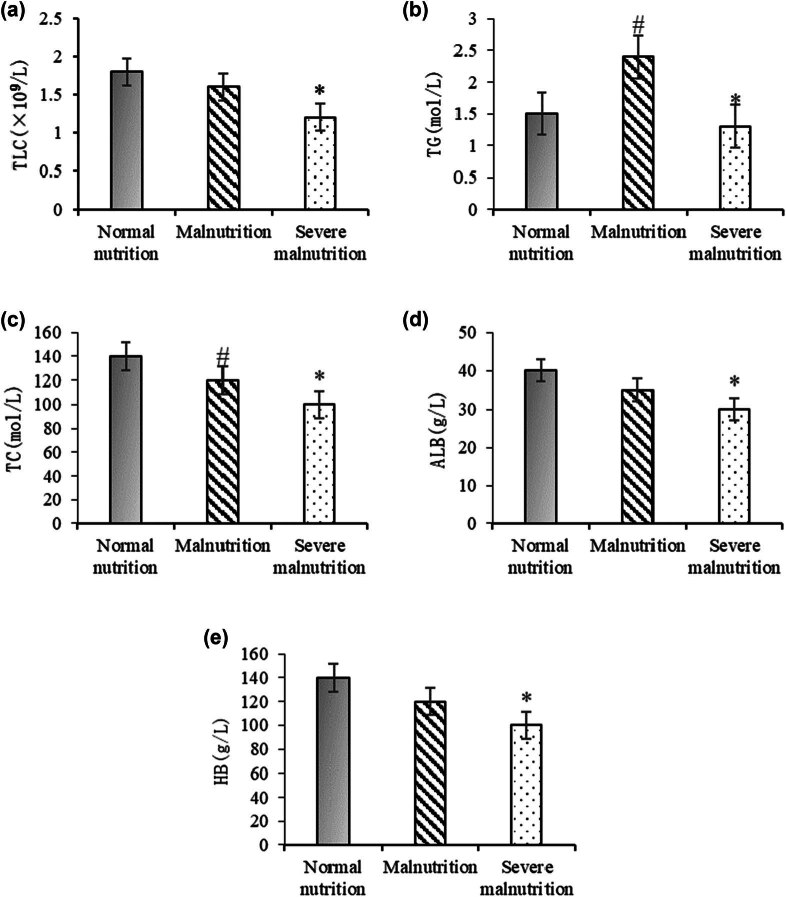
Comparison of biochemical indicators in children with different nutritional statuses. Note: (a) TLC; (b) TG; (c) TC; (d) ALB; and (e) Hb. *A significant difference between the severe malnutrition group and the normal nutrition group (*P* < 0.05). #A significant difference between the malnutrition group and the normal nutrition group (*P* < 0.05).

Biochemical testing was conducted on all children in the intravenous and oral groups, as depicted in Figure 1. In Figure 1a, the normal nutrition group exhibited the highest TLC levels, followed by the malnutrition group, with the severe malnutrition group showing the lowest levels. The severe malnutrition group showed a significant difference compared to the normal nutrition group (*P* < 0.05), and the malnutrition group also showed a significant difference compared to the normal nutrition group (*P* < 0.05). In Figure 1b, the malnutrition group had the highest TG levels, followed by the normal nutrition group, with the severe malnutrition group showing the lowest levels. There was a significant difference between the malnutrition and normal nutrition groups (*P* < 0.05) and a significant difference between the severe malnutrition and normal nutrition groups (*P* < 0.05). In Figure 1c, the normal nutrition group had the highest TC levels, followed by the malnutrition group, with the severe malnutrition group showing the lowest levels. Significant differences were found between the malnutrition and normal nutrition groups (*P* < 0.05) and between the severe malnutrition and normal nutrition groups (*P* < 0.05). In Figure 1d, the normal nutrition group exhibited the highest ALB levels, followed by the malnutrition group, with the severe malnutrition group showing the lowest levels. There was a significant difference between the severe malnutrition and normal nutrition groups (*P* < 0.05), while no significant difference was observed between the malnutrition and normal nutrition groups (*P* > 0.05). In Figure 1e, the normal nutrition group had the highest ALB levels, with the severe malnutrition group showing the lowest levels. A significant difference was observed between the severe malnutrition and normal nutrition groups (*P* < 0.05).

### 3.5 Comparison of kidney function indicators

**Figure 2:**
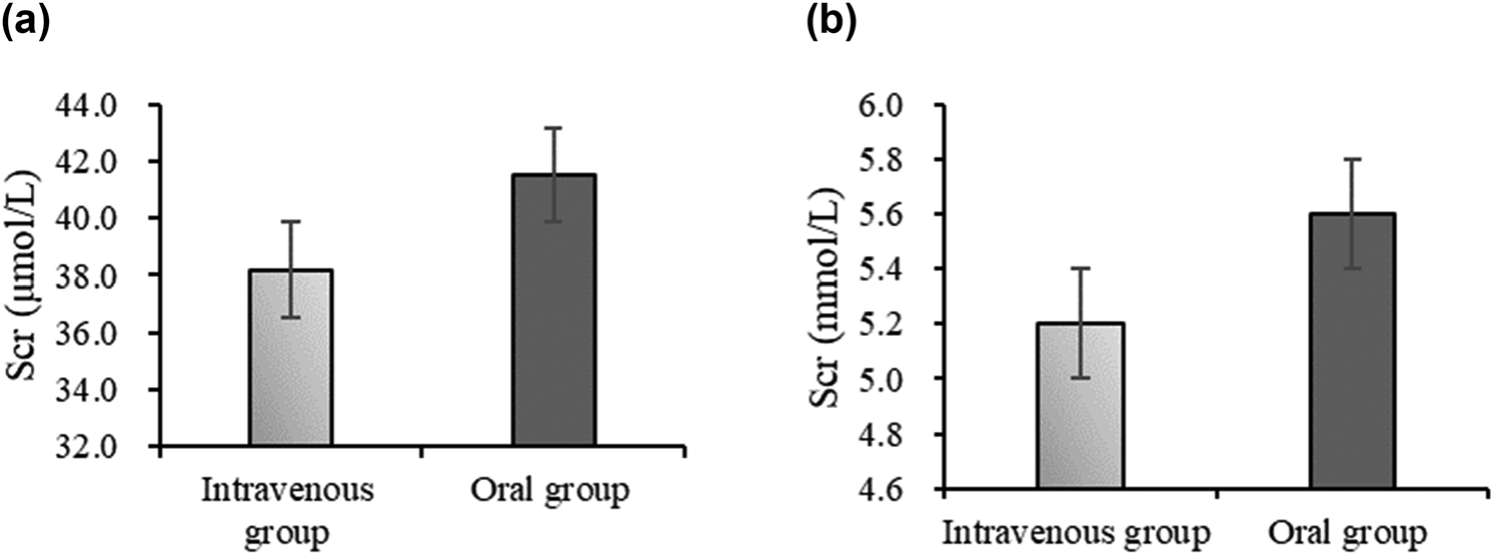
Comparison of kidney function indicators. Note: (a) Scr and (b) BUN.

Figure 2 shows that in Figure 2a, the average Scr for the intravenous group was 38.2 ± 12.1 μmol/L, and for the oral group, it was 41.5 ± 14.3 μmol/L. There was no statistically significant difference between the two groups (*P* > 0.05). In Figure 2b, the average BUN for the intravenous group was 5.2 ± 1.8 mmol/L, and for the oral group, it was 5.6 ± 2.1 mmol/L, both within the normal range. There was no statistically significant difference between the two groups (*P* > 0.05).

### 3.6 Impact of different surgery types on postoperative complications

The incidence of DGC in the gastrointestinal surgery group was significantly higher than in the other groups (52.4% vs 28.6%, *P* = 0.03). The endocrine-related surgery group had a higher risk of infection (14.3% vs 4.8%, *P* = 0.08), but this difference did not reach statistical significance. Surgical complexity (Clavien–Dindo classification) was positively correlated with the degree of malnutrition (*r* = 0.34, *P* = 0.01).

### 3.7 Surgery conditions and postoperative outcomes

Both groups of children had good surgical outcomes, and the postoperative recovery and complication occurrences were presented in Table 6. In the intravenous group, the number of cases with infection, DWH, DGC, and RSC were 3 (10%), 6 (20%), 12 (40%), and 1 (3.33%), respectively. In the oral group, the number of cases of infection, DWH, and DGC was 2 (6.67%), 6 (20%), and 9 (30%), respectively.

**Table 6:** Comparison of poor prognosis of children after surgery

Group	Items			
	Infection	DWH	DGC	RSC
Intravenous group	3 (10%)	6 (20%)	12 (40%)	1 (3.33%)
Oral group	2 (6.67%)	6 (20%)	9 (30%)*	0

^*^Suggested a statistical remarkable difference with *P* < 0.05.

Variance analysis in Table 7 indicated that there were no significant differences in the incidence of all complications between the two groups (*P* > 0.05), although the incidence of DGC showed an increasing trend in the intravenous group (40% vs 30%). Further subgroup analysis revealed that the risk of DGC in children undergoing gastrointestinal surgery was significantly higher than in those undergoing other types of surgery (*P* = 0.03).

**Table 7:** Results of between-group variance analysis for postoperative complications

Complication	Intravenous group (*n* = 30)	Oral group (*n* = 30)	*F*	*P*
Infection	3 (10%)	2 (6.67%)	0.21	0.65
DWH	6 (20%)	6 (20%)	0.00	1.00
DGC	12 (40%)	9 (30%)	0.67	0.41
RSC	1 (3.33%)	0	1.02	0.31

### 3.8 Changes in glucose of children before and after surgery

**Figure 3:**
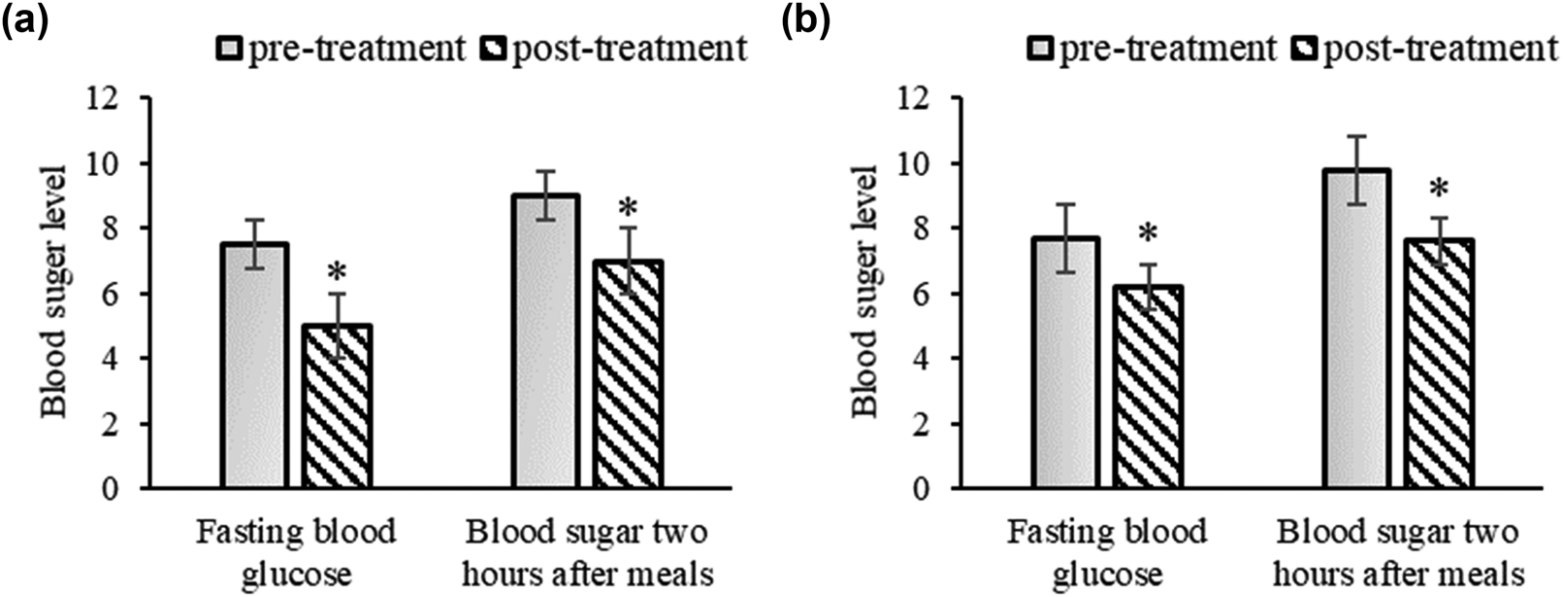
Changes in glucose of children before and after surgery. Note: (a) intravenous group; (b) oral group. *Suggested a significant difference with *P* < 0.05 based on the preoperative level.

As depicted in Figure 3, it was evident that after the surgery, all children experienced varying degrees of reductions in FBG levels and post-meal blood glucose levels. These reductions may be attributed to the metabolic demands of surgery and the healing process of the surgical wounds. In Figure 3a, the FBG levels after treatment were significantly lower than before treatment (*P* < 0.05). The blood sugar levels 2 h after meals were also significantly lower after treatment compared to before treatment (*P* < 0.05). In Figure 3b, the FBG levels after treatment were significantly lower than before treatment (*P* < 0.05). The blood sugar levels 2 h after meals were significantly lower after treatment compared to before treatment (*P* < 0.05). Clearly, postoperatively, all children exhibited varying degrees of reduction in both FBG and PBG levels. These reductions may be attributed to the metabolic demands of surgery and the wound-healing process.

### 3.9 Multivariate logistic regression analysis results

Through the analysis of all the data in conjunction with the duration and outcomes of the children’s surgeries, some important findings have emerged. First, it was found that the FBG levels below 6.5 mmol/L (odds ratio [OR] = 2.4, 95% confidence interval [CI]: 1.4–5.1) and BMI below 17.3 (OR = 2.7, 95% CI: 1.6–5.72) were independent risk factors affecting nutritional support. A surgery duration of ≥120 min (OR = 1.8, 95% CI: 1.1–3.0) was also identified as an independent risk factor for the effectiveness of nutritional support. Postoperative complications (such as infection) did not reach statistical significance due to insufficient sample size (*P* = 0.08). These findings suggested that excessively low glucose levels and BMI may lead to suboptimal effectiveness of nutritional support.

## 4 Discussion

Through a comprehensive assessment of the nutritional status of children with congenital diabetes before, during, and after surgery, this work identified a significant nutritional risk in this specific patient population during the surgical period. First, the preoperative nutritional status exerted a crucial role in the recovery and rehabilitation after surgery. This work observed that many children with congenital diabetes patients had malnutrition before surgery, such as low body weight and insufficient protein intake. These factors may hinder the postoperative recovery process and increase the risk of infection and complications. Second, nutritional support during surgery also significantly influenced the surgical outcomes. During surgery, children with congenital diabetes patients underwent anesthesia and surgical trauma, leading to an increased metabolic rate and greater nutritional demands. Therefore, an appropriate nutritional support strategy was essential for maintaining metabolic balance and ensuring the success of the surgery. Finally, postoperative nutritional support was of paramount importance for the recovery and growth of children with congenital diabetes patients.

The reduction in postoperative blood glucose levels is the result of multiple factors acting in concert. In addition to the metabolic demands associated with surgical trauma and wound healing, the following factors may influence blood glucose fluctuations. Some children may experience exacerbated insulin resistance due to the use of glucocorticoids or β-receptor blockers for pain management [18]. Anesthetics used during surgery (such as propofol) may transiently lower blood glucose by inhibiting hepatic gluconeogenesis. Surgical stress leads to elevated cortisol and catecholamine levels, which temporarily increase glycogenolysis, but prolonged stress may deplete liver glycogen stores, resulting in delayed postoperative hypoglycemia [16,19]. In this study, the infection rate in the intravenous group was 10%, and infection-related inflammatory factors (such as IL-6) could interfere with insulin signaling pathways, exacerbating blood glucose fluctuations. The intravenous group’s reliance on glucose infusion may mask the true metabolic demands, whereas the oral group experienced insufficient energy intake due to postoperative fasting or impaired digestive function. Future studies should comprehensively analyze blood glucose regulation mechanisms through dynamic continuous glucose monitoring combined with blood biochemical markers (such as cortisol and inflammatory factors). It is important to note that although the PPNRA scale can reflect the common characteristics of perioperative nutritional risk, its applicability in children with congenital diabetes still requires further validation. For example, the scale does not include diabetes-specific metabolic indicators (such as glucose variability coefficient and insulin requirements), which may lead to an underestimation of the nutritional risk in these patients. Future studies should focus on optimizing the existing assessment system by incorporating diabetes-specific parameters.

In recent years, several studies explored the challenges of perioperative management in children with diabetes. For instance, a prospective cohort study by Wang et al. [20] found that the preoperative HbA1C > 7.850% is associated with an increased incidence of surgical site infections, thereby providing some support for the notion that higher preoperative HbA1c levels are correlated with an elevated risk of postoperative infections. In contrast, in this study, FBG < 6.5 mmol/L was identified as an independent risk factor, suggesting that different dimensions of glucose control (acute vs chronic) may have differential effects on outcomes. Rossing et al. [21] indicated that chronic kidney disease is a common complication of type 2 diabetes (T2D). Glucagon-like peptide-1 receptor agonists can improve blood glucose control and reduce weight in T2D patients, with some of them reducing the risk of cardiovascular (CV) events in patients with high CV risk. Nelson and Sumpter [22] noted that both surgery and anesthesia trigger stress responses, leading to significant metabolic changes in patients, thereby increasing blood glucose variability. Close monitoring of glucose levels and a clear insulin administration protocol can help mitigate these characteristic responses. Martin et al. [23] reported that in the United States, approximately 1 in every 300 children is affected by T1D. In the perioperative period, careful attention should be given to appropriate insulin administration, as well as the management of hypoglycemia and hypertension following anesthesia and surgery. However, compared to the aforementioned studies, the strength of this research lies in its systematic analysis of the nutritional risk characteristics of children with congenital diabetes, a unique subgroup, particularly the high incidence of severe malnutrition (36.67%), providing clinical evidence for targeted interventions. Future studies should integrate metabolic indicators (such as ketone levels) and dynamic glucose monitoring data to more comprehensively assess risk. This study, based solely on a grouping strategy for nutritional support methods, may overly simplify the interaction between surgical stress, metabolic demands, and nutritional status. For example, children undergoing major gastrointestinal surgery may inherently require extended intravenous support, while minor surgeries may be more suited to oral intake. Subsequent research should stratify participants based on surgical complexity and disease severity to refine nutritional support strategies.

The postoperative recovery period is a critical time for children’s growth and development, and malnutrition can lead to growth retardation and weakened immune function. Compared to nutritionally normal patients, children with diabetes exhibit a significant decrease in total lymphocyte count, which serves as a representative marker of their immune status, raising significant concerns. Over time, this may increase the risk of complications and other infections. Therefore, it is crucial to develop a reasonable nutritional support plan to meet the energy and nutritional needs of children with congenital diabetes, promoting their recovery and growth [24,25]. Recommendations for implementing individualized nutritional support strategies: according to the WHO energy requirements formula for children, the recommended perioperative intake is resting energy expenditure (REE) × 1.3–1.5, where REE (kcal/day) = 22 × body weight (kg) + 500 (for children aged 6–12 years). Postoperative protein requirements in children with congenital diabetes are increased, with a recommended daily intake of 1.5–2.0 g/kg, prioritizing high-biological-value proteins (such as whey protein and casein). Carbohydrates should account for 45–50% of total energy intake, with a focus on low glycemic index (<55) foods (such as whole wheat bread and oats), provided in 5–6 meals to avoid significant blood glucose fluctuations. Fat should constitute 30–35% of total energy, with an emphasis on monounsaturated fatty acids (such as olive oil) and limiting saturated fats (<10%). Capillary blood glucose should be monitored every 4 h, with adjustments made based on the readings. If FBG > 10 mmol/L, the glucose infusion rate should be reduced (e.g., from 5 to 3 mg/kg/min), and insulin dosages should be adjusted accordingly. For children with infections or DWH, additional supplementation with vitamin C (200 mg/day) and zinc (10 mg/day) is recommended to promote wound healing. Between 24 and 48 h postoperatively, a gradual transition from intravenous nutrition to enteral nutrition should be made, starting with elemental formulas (e.g., Peptamen Junior), and transitioning to polymeric formulas once well-tolerated [26,27].

Although the sample size of 60 children in this study is sufficient for an initial understanding, it limits the statistical power to detect subtle associations and restricts the generalizability to a broader population. Small sample sizes increase the risk of Type II errors, particularly in subgroup analyses (e.g., severe malnutrition vs normal nutrition). To address this limitation, future plans include a larger, multicenter cohort study aimed at recruiting at least 200 participants. This expansion will enhance the robustness of risk factor identification (e.g., BMI < 17.3 as an independent predictor) and allow for stratified analyses based on surgical type and disease severity. Due to the specificity of children with congenital diabetes during the surgical period, there are inherent challenges, including potential ethical and operational limitations. Additionally, the retrospective nature of this study introduces inherent limitations, including potential biases from incomplete records or unmeasured confounding factors (e.g., variations in preoperative care). For instance, dietary intake data relied on 24-h recalls from medical charts, which may not fully capture habitual nutritional patterns. Although the 24-h dietary recall is a commonly used clinical nutritional assessment tool, its reliance on caregivers’ recollection may either underestimate or overestimate actual intake. This study partially addressed this limitation by combining the FFQ with biochemical indicators, but future studies should incorporate more objective methods (e.g., doubly labeled water or smart dietary tracking devices) to improve data reliability. The development and implementation of nutritional support plans need to consider various factors, such as the type of surgery, the nutritional status of the children, surgical risks, and more. Therefore, further research and exploration are required to determine the optimal nutritional support strategies.

## 5 Conclusion

This study highlights the importance of PPNRA and support in children with congenital diabetes. However, there are several limitations to the study: first, the small sample size (*n* = 60) and the single-center retrospective design may limit the generalizability of the conclusions; second, the PPNRA scale has not been optimized for the metabolic characteristics of diabetes, which may result in an underestimation of risk. Therefore, clinical implementation should be approached with caution, and it is recommended to verify the findings using specialized assessment tools. Future research should expand the sample size through multicenter collaboration and adopt a prospective design to monitor blood glucose and nutritional indicators in real time. In addition, exploring personalized nutritional support strategies (such as dynamic adjustments based on continuous glucose monitoring) and the application of metabolomics in nutritional interventions will help optimize perioperative management for these children.
